# Digital competencies in medical education in Switzerland: an overview of the current situation

**DOI:** 10.3205/zma001355

**Published:** 2020-11-16

**Authors:** Stefanie C. Hautz, Michele Hoffmann, Aristomenis K. Exadaktylos, Wolf E. Hautz, Thomas C. Sauter

**Affiliations:** 1 Universität Bern, Universitätsspital Bern, Inselspital, Universitäres Notfallzentrum, Bern, Switzerland

**Keywords:** digital transformation, digital competencies, medical education, Switzerland

## Abstract

**Background: **Today’s medical students are growing up in a digital age in which the use of smartphones and smart devices is now irrevocably part of professional life. However, the abilities to use these devices that have become so ubiquitous in private life can only be partially transferred to work with patients and the medical setting. Since little is known about digitalization in medical education, the aim of this paper is to gain an overview of the current training in digital competencies at Swiss universities.

**Methods: **The medical deans at all Swiss universities were contacted by telephone and informed about an online survey. The invitation to fill out the Survey Monkey questionnaire was subsequently sent by email to the specific contacts at each university. The survey consisted of questions to be answered using a defined scale and open-ended questions. The survey’s focus, topics involving digital competencies, is based on the content in the Principal Relevant Objectives and Framework for Integrative Learning and Education in Switzerland (PROFILES) and the National Competency-based Catalogue of Learning Objective in Undergraduate Medicine (NKLM).

**Results**: All of the dean’s offices that were contacted participated in the survey. The topics on digitalization were all rated as relevant or very relevant. Our survey shows a heterogeneous picture in terms of implementing PROFILE and NKLM content. A few universities have well-established educational approaches or even implemented curricula, but often these are still in development. Participants also mentioned factors that are necessary for successfully setting up and implementing curricula dealing with digitalization and factors that can impede such efforts.

**Conclusion: **The importance of acquiring digital competencies during medical study is known and recognized by all Swiss medical schools. Curricular integration varies in its progress and represents major challenges for the medical faculties. It is precisely the inclusion of students in such efforts that may be a potential response to this challenge.

## Zusammenfassung

**Hintergrund: **Die derzeitigen Medizinstudierenden wachsen in einem digitalen Zeitalter auf, aus dem der Umgang mit Smartphones und Smart Devices im beruflichen Alltag nicht mehr wegzudenken ist. Die Fähigkeiten im Umgang mit diesen Devices, die im privaten Alltag wie selbstverständlich angewendet werden, lassen sich jedoch nur teilweise auf die Arbeit mit Patienten und das medizinisch-professionelle Umfeld übertragen. Da bisher wenig zum Thema Digitalisierung im Medizinstudium bekannt ist, ist das Ziel dieser Arbeit, einen Überblick über die derzeitige Ausbildung in digitalen Kompetenzen an den Universitäten der Schweiz zu erhalten.

**Methoden: **Die medizinischen Dekanate aller Schweizer Universitäten wurden telefonisch kontaktiert, mit dem Hinweis auf eine Online Umfrage. Die Einladung für diese in Survey Monkey implementierte Umfrage wurde im Anschluss per E-Mail an die entsprechenden Ansprechpartner verschickt und bestand aus einem Skalenanteil und Freitextfragen. Der in der Umfrage abgefragte Themenkomplex „digitale Kompetenzen“ orientiert sich an den Inhalten, wie sie im Principal Relevant Objectives and Framework for Integrative Learning and Education in Switzerland (PROFILES) und dem Nationalen Kompetenzbasierten Lernzielkatalog Medizin (NKLM) genannt werden.

**Ergebnisse:** Alle befragten Dekanate haben an der Umfrage teilgenommen. Die genannten Themen aus dem Bereich der Digitalisierung werden allesamt als relevant oder sehr relevant bewertet. Unsere Umfrage zeigt ein heterogenes Bild bezüglich der Umsetzung dieser Inhalte. An wenigen Universitäten ist eine gut etablierte oder sogar curricular implementierte Ausbildung vorhanden, oft aber ist sie im Aufbau. Zusätzlich benannten die Teilnehmer förderliche und hinderliche Faktoren bezüglich des Aufbaus und der Umsetzung curricularer Implementation zum Thema Digitalisierung.

**Schlussfolgerung: **Die Bedeutung des Erwerbs digitaler Kompetenzen im Rahmen des Medizinstudiums ist allen medizinischen Fakultäten in der Schweiz bekannt. Die curriculare Integration ist unterschiedlich weit fortgeschritten und stellt die Fakultäten vor große Herausforderungen. Gerade der Einbezug Studierender in die entsprechende Lehre scheint eine mögliche Antwort auf diese Herausforderung zu sein.

## Background

How doctors provide treatment to their patients is a process that is subject to changing times. Over the last decade learning and working in medicine has been increasingly influenced by digital tools, and the “digital transformation” is now a popular topic. Regarding digitalization in medicine, the World Economic Forum, for example, is assuming that not just the use of new media will change the future healthcare system, but is also a decisive change in the transition to patient-centered healthcare that enables citizens to take much more responsibility for their healthcare and that of their families [1]. The US Food and Drug Administration (FDA) states that the scope of digital healthcare encompasses mobile health (mHealth), health information technology (IT), wearable devices, telehealth and telemedicine and personalized medicine [https://www.fda.gov/medical-devices/digital-health-center-excellence]. Switzerland has also taken up the topic of change in medicine through the possibilities offered by digitalization. Starting in 2020 all Swiss medical providers will be required to compile therapeutic and diagnostic records into an electronic patient medical chart and – if a patient has such a chart – to take the information contained in it into consideration when making medical decisions [2].

However, the existence of digital tools such as electronic patient records is only half of the story; how to handle such tools is the other. What this entails has not yet been defined or standardized. Accordingly, there is little data on the risks and benefits that come with this transformation and the need for education and training is largely unknown or unspecified. The few defined terms that exist for this kind of “health literacy” are rather generic in nature. The WHO, for instance, defines them as “the cognitive and social skills which determine the motivation and ability of individuals to gain access to, understand and use information in ways which promote and maintain good health” [3]. So it is not surprising that healthcare researchers have long criticized the divide between health and education [4]. This also applies to the German-speaking countries, as Haag et al. remark when they state that medical students have not been sufficiently prepared to cope with the challenges of digital medicine [5].

Although students studying medicine today have grown up in a digital age in which the use of smartphones, apps and smart devices is ubiquitous and irreversibly part of daily life, and the ability to use such devices goes unquestioned in private life, such abilities are, however, only partially transferrable to work with patients and the professional medical setting [6]. Mobile apps, such as digital decision aids, scoring systems and digital triage tools, are available for smartphones and have now become fixed components of routine work. In the face of this ever-present availability of knowledge on the internet, the study of medicine must be adapted and changed from textbook-based teaching to process-based instruction (“from textbooks to Dr. Google”). This change is not just taking place for medical students and physicians-to-be, but also for patients, in that informed patients like to communicate with doctors on equal footing, wishing to have their knowledge put into context and explained. Such patients require doctors to use communication strategies that must first be acquired. For example, a web-based symptom checker currently reports more than 15 million visits monthly [7] and more than a third of Americans attempt to diagnose themselves using internet sources [8]. Kickbusch consequently states that digital literacy is becoming increasingly more important, not just in education, but also for patients and society itself [4]. The situation is no different in respect to legal aspects (e.g. data protection or digital communication between patients). These must also be discussed anew [9]. This is not surprising since only around half of the WHO countries provide specific protection of the private sphere for personal health information [10]. Work is being done at the European level to create uniform data protection guidelines for the administrative handling of personal health information [11].

In summary, it can be said that digitalization is now recognized as part of routine medical practice. Now, the institutions of higher education must come to grips with it. To facilitate this, Switzerland has begun an initiative, e-Health-Strategie 2.0, in which it identifies different areas of action, one of them being the enabling of digitalization. It recognizes the nation-wide lack of skills in this area and emphasizes the importance of digital competencies as a major goal [2]. A mandatory, specifically designed set of courses will be necessary to transfer this area of action to education [12]. While the two relevant catalogues of learning objectives – the Principal Relevant Objectives and Framework for Integrative Learning and Education in Switzerland (PROFILES) [13] and the National Competency-based Catalogue of Learning Objectives in Undergraduate Medicine (NKLM) [http://www.nklm.de] – offer a basis for this enormous undertaking, they can only partly offer detailed help to individual institutions, because, while they do identify competencies such as telemedical aftercare and treatment of chronic diseases, they hardly (if at all) break these topics down into concrete learning objectives.

Parallel to this, the topic of digitalization has also been taken up by the Swiss Medical Association (FMH) in the ambulant sector and it has been determined that the opportunities presented by digitalization can only be optimally realized if the digital competencies of physicians are fostered in tandem with those of patients. All of these approaches emphasize the immense importance of adapting post-graduate training and continuing medical education to address digitalization and defining this as a major strategic task [14].

Little is known about the current situation in Switzerland concerning the topic of digitalization in undergraduate medical education. The aim of this survey was therefore to gain an overview of the current situation involving training in digital competencies at Swiss universities and, as a result, to give the medical faculties a chance to learn from each other.

## Methods

Between August 20 and September 20, 2019, the dean’s offices at all seven Swiss universities were contacted by telephone to inform them of an online survey. The invitation to participate in the Survey Monkey questionnaire was then sent by email to the specific contacts. Those contacted directly by email were study program coordinators within the dean’s offices.

The survey’s focus on the topic of digital competencies was based on how telemedicine is handled in PROFILES [12], NKLM [15] and the pilot program implemented by von Kuhn et al. [16] at the University of Mainz, Germany, which was published about in 2018.

The survey encompasses a rated section (1=lowest rating; 3=highest) and an opportunity to freely respond to open questions. The scales are reported here with their absolute values due to the small number of cases. The written responses were systematically analyzed and clustered. Inductive categories according to Mayring were created to the extent it was possible using the few responses [17]. To do this, the responses regarding supportive and hindering factors were grouped by topic to form four categories (see table 1 (Tab. 1)).

The topics about which the universities were asked regarding their course offerings cover: secure digital communication (from email to Whatsapp), social media (doctors on Twitter and rating portals), telemedical aftercare and care of chronic diseases, medical apps and smart devices, use of digital content (Uptodate, Compendium, etc.), telemedical (emergency) care (e.g. teleradiology or dermatology), virtual reality-/augmented reality-supported training or treatment, and legal and ethical aspects (data protection, personal privacy). The original questions are in attachment 1 .

Because there are four official languages spoken in Switzerland, of which three are used in higher education (German, French and Italian), we chose to conduct the survey in English.

## Results

All seven Swiss universities that offer traditional bachelor and master degree programs in medicine participated in the survey (response rate 100%). Also included in the survey was ETH Zürich, which offers a bachelor degree program that strongly connects medicine and the technical sciences within the context of human health in its medical curriculum [https://ethz.ch/en/studium/bachelor/studienangebot/systemorientierte-naturwissenschaften/medizin.html]. The surveyed universities have consented to the publication of the aggregate data.

### 1. Current situation regarding the topics and their importance as estimated by the Swiss medical faculties

The topics currently offered at the universities are listed in table 1 (Tab. 1) and were rated by the survey participants according to their importance.

#### 2. The existing course offerings

At five universities (62.5%) the curricular content was not embedded in explicit courses, but rather taught across the curriculum in an interdisciplinary manner. 

Two universities have courses for which eight ETCS credits are given; one university has a course with one 1 ETCS credit. 

One course on the topic of telemedicine has been in existence since 2008. Another course on the topic of digital health/data science has been offered since 2010, and a course entitled Digital Medicine will be offered for the first time in 2019.

All courses explicitly referred to are mandatory.

Responses to the question about what the ideal curriculum could look like included mapping the “digital reality” with practical elements and the teaching and learning of a critical approach to and handling of digitalization.

According to the survey, exam-relevant aspects of digital competencies are mainly to have a critical approach and the ability of students to deal with new methods or technical opportunities critically and in a reflective manner and to ascertain the social context.

The teaching methods that are employed at the universities were listed: introductory lectures, hands-on workshops (e.g. simulations), small-group instruction, e-learning and assigned projects, or specific digital formats such as hackathons (e.g. creation of a software product within a limited period of time), massive open online courses (MOOC), and forums.

#### 3. Supportive and hindering factors

The beneficial and adverse factors identified in the survey are presented in table 2 (Tab. 2).

Time and money and the importance of having a competent coordinator or the support of IT specialists were primarily cited as necessary factors for improved implementation.

## Discussion

All of the Swiss medical faculties rated the topics dealing with digitalization mentioned here as being relevant or very relevant. Our survey shows a heterogeneous picture in terms of implementing this content. A few universities have well-established educational approaches or even implemented curricula, but often these are still in development.

The reason why there has been hardly any course offerings on this topic appears mainly to do with four factors: lack of support in the development and implementation of such courses, lack of staff for the coordination of planning such courses, the appropriate budget, and, primarily, the absence of curricular content. 

The two existing catalogues of learning objectives do not seem to cover the need in this area adequately. Learning objectives that are directly transferrable to practice and provide direction in education are used to familiarize students with the urgently needed skills in health literacy [4]. 

The diversity of the teaching formats appears to be viewed as less of a problem. An extremely wide variety was mentioned and also used to impart the new content.

A major challenge for the curricular implementation of digital topics, however, appears to be the required three-fold expertise in the digital world, medical education, and medical science. Not every medical faculty seems to be able to fully meet this requirement. Pilot projects, such as the curriculum implemented by Kuhn et al., can make valuable contributions and serve as blueprints for other universities [16].

In terms of co-development, the inclusion of students as both the target group and digital content experts could be done in the context of a peer teaching program. This offers a counterargument to the assumption that rising costs automatically accompany this topic. In addition to rebutting concerns over budgets, peer teaching could also counteract both the rapid transformation of digital content and its potential obsolescence, thus providing a resource-saving, up-to-date and motivating approach to implementation [18].

Furthermore, the empowerment of students to critically approach and use digital opportunities offers a way to deal with this challenge. This rather basic critical approach was heavily favored in our survey over the teaching of specific content and appears to be almost dictated by the rapid transformation in which any knowledge taught at the beginning of medical study already seems obsolete to students. It is unknown if digital competence and critical thinking are skills which can be taught or learned at all independently of the specific context or if they must be embedded in the context of medical expertise. Whether they are or not requires scientific verification. As with the knowledge gained from research on problem-based learning, it could be that the acquisition of generic skills is difficult or impossible [19]. Another open question is the appropriate time point at which to teach this content. It is probable that most students have their first direct contact with courses on digital topics during the clinical work in their final year of study. Placing peer teaching or courses during this study phase could be helpful.

Interdisciplinary networking among medical teachers also offers a possible strategy to deal with the challenge of needing a large amount of resources. Similar digital competencies are being required of all those in medical occupations, from pharmacists and emergency medical technicians to nurses. Networking would also spread out the burden of needing to have the necessary expertise and ensure the inclusion of competence from the widest range of different specializations.

## Conclusion

The importance of acquiring digital competencies during medical study is known and recognized by all Swiss medical schools. Curricular integration varies in its progress and represents major challenges for the medical faculties. It is precisely the inclusion of students in such efforts that may be a potential response to this challenge.

## Acknowledgements

The authors wish to thank those who participated in the survey for promptly responding to the questions.

## Competing interests

The authors declare that they have no competing interests. 

## Supplementary Material

Original questions

## Figures and Tables

**Table 1:**
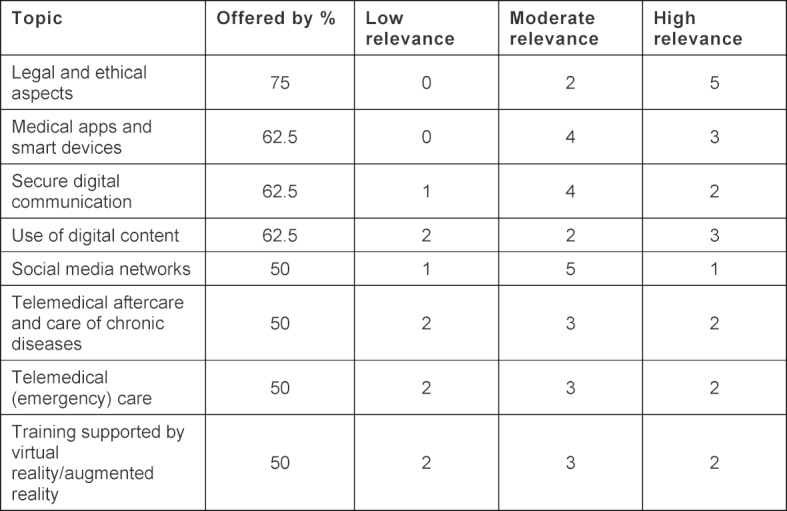
Current situation regarding the topics and their importance as estimated by the Swiss medical faculties, categorized by importance

**Table 2:**
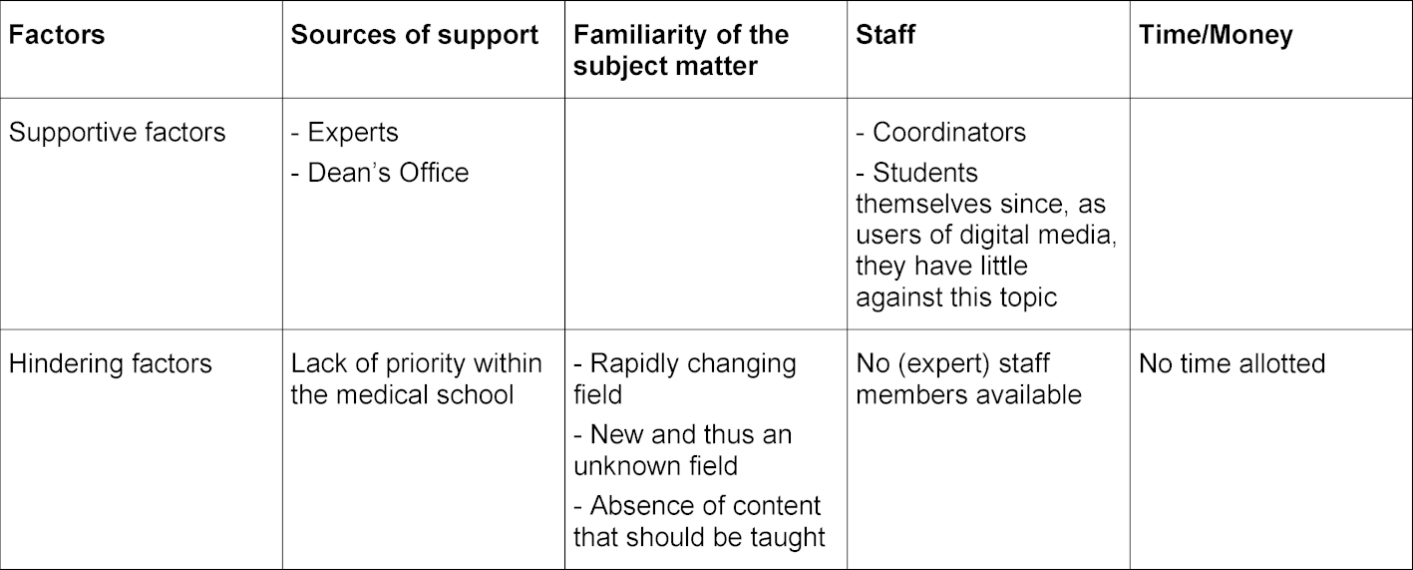
Supportive and hindering factors
