# Supplementary material for: Digital competencies in medical education in Switzerland: an overview of the current situation
Source: GMS J Med Educ. 2020 Nov 16;37(6):Doc62. doi: 10.3205/zma001355 (PMC7672378; doi:10.3205/zma001355)
Supplement: Original questions [file JME-37-62-s-001.pdf]

1. **Which of these topics are taught or planned to be included in the curriculum of your university in the field of "Digital Health"?**

   Secure Digital Communication (from Email to Whatsapp)

   Social networks (the doctor on Twitter, rating portals)

   Telemedical aftercare and care of chronic diseases

   Medical Apps or Smart Devices

   Usage of Digital Health Services (Uptodate, Compendium, etc.)

   Telemedical (emergency) treatment (teleradiology/dermatology, etc.)

   Virtual Reality/Augmented Reality supported training and treatment

   Legal and ethical aspects (data protection, personal rights)

   Digital health / Data science

2. **Which university courses do you currently offer?**

   Name of the course

   Duration (hours)

   Target group

   Mandatory or voluntary?

   Is there an evaluation?

   First year of implementation?

   By whom is the subject taught?

Attachment 1 to: Hautz SC, Hoffmann M, Exadaktylos AK, Hautz WE, Sauter C. *Digital competencies in medical education in Switzerland: an overview of the current situation.* GMS J Med Educ. 2020;37(6):Doc62. DOI: 10.3205/zma001355

3. **Please rate the importance of the listed topics at your university (1 is the worst rating, 3 the best rating)**

   Secure Digital Communication (from Email to Whatsapp)

   Social networks (the doctor on Twitter, rating portals)

   Telemedical aftercare and care of chronic diseases

   Medical Apps or Smart Devices

   Usage of Digital Health Services (Uptodate, Compendium, etc.)

   Telemedical (emergency) treatment (teleradiology/dermatology, etc.)

   Virtual Reality/Augmented Reality supported training and treatment

   Legal and ethical aspects (data protection, personal rights)

   Digital health / Data science

4. **What are hindering factors which you have encountered in the transfer of knowledge in the field of "digital medicine"?**
5. **What are supporting factors which you have encountered in the transfer of knowledge in the field of "digital medicine"?**
6. **What would the ideal Curriculum "Digital Medicine" look like for you?**
7. **What kind of support would you like to have in order to better represent the topic of digital health in your University?**
8. **Are there exam-relevant topics in the field of digital Health?**
9. **Which didactic methods / teaching formats do you use in the field of "digital health"?**
